# An integrative approach for exploring the nature of fibroepithelial neoplasms

**DOI:** 10.1038/s41416-022-02064-2

**Published:** 2022-12-16

**Authors:** Jihui Yun, Woohang Heo, Eun-Shin Lee, Deukchae Na, Wonyoung Kang, Jinjoo Kang, Jeesoo Chae, Dakyung Lee, Woochan Lee, Jinha Hwang, Tae-Kyung Yoo, Bok Sil Hong, Hye-Youn Son, Dong-Young Noh, Charles Lee, Hyeong-Gon Moon, Jong-Il Kim

**Affiliations:** 1 Genomic Medicine Institute, Medical Research Center, Seoul National University, Seoul, Republic of Korea; 2 Department of Biomedical Sciences, Seoul National University College of Medicine, Seoul, Republic of Korea; 3 Interdisciplinary Program on Tumor Biology, Seoul National University College of Medicine, Seoul, Republic of Korea; 4 Department of Surgery, Seoul National University Hospital, Seoul, Republic of Korea; 5 Ewha Institute of Convergence Medicine, Ewha Womans University Mokdong Hospital, Seoul, Republic of Korea; 6 The Jackson Laboratory for Genomic Medicine, Farmington, Connecticut USA; 7 Department of Life Sciences, Ewha Womans University, Seoul, Republic of Korea; 8 Center for Medical Innovation, Seoul National University Hospital, Seoul, Republic of Korea; 9 Cancer Research Institute, Seoul National University, Seoul, Republic of Korea; 10 Department of Surgery, Seoul National University College of Medicine, Seoul, Republic of Korea; 11 Precision Medicine Center, The First Affiliated Hospital of Xi’an Jiaotong University, Xi’an, People’s Republic of China; 12 Department of Biochemistry and Molecular Biology, Seoul National University College of Medicine, Seoul, Republic of Korea

**Keywords:** Breast cancer, Cancer genomics

## Abstract

**Background:**

Malignant phyllodes tumour (MPT) is a rare breast malignancy with epithelial and mesenchymal features. Currently, there are no appropriate research models or effective targeted therapeutic approaches for MPT.

**Methods:**

We collected fresh frozen tissues from nine patients with MPT and performed whole-exome and RNA sequencing. Additionally, we established patient-derived xenograft (PDX) models from patients with MPT and tested the efficacy of targeting dysregulated pathways in MPT using the PDX model from one MPT.

**Results:**

MPT has unique molecular characteristics when compared to breast cancers of epithelial origin and can be classified into two groups. The PDX model derived from one patient with MPT showed that the mouse epithelial component increased during tumour growth. Moreover, targeted inhibition of platelet-derived growth factor receptor (PDGFR) and phosphoinositide 3-kinase (PI3K)/mammalian target of rapamycin (mTOR) by imatinib mesylate and PKI-587 showed in vivo tumour suppression effects.

**Conclusions:**

This study revealed the molecular profiles of MPT that can lead to molecular classification and potential targeted therapy, and suggested that the MPT PDX model can be a useful tool for studying the pathogenesis of fibroepithelial neoplasms and for preclinical drug screening to find new therapeutic strategies for MPT.

## Background

Breast fibroepithelial tumours, including fibroadenomas and phyllodes tumours, are biphasic neoplasms characterised by proliferation of epithelial and stromal cells of the mammary glands [1]. Phyllodes tumours comprise <1% of all primary breast neoplasms, and up to one-third of phyllodes tumours are classified as malignant phyllodes tumours (MPTs) based on histologic findings, such as degree of mitosis and extent of stromal growth [1]. Up to 67% of patients with MPT develop local recurrences, and 21% experience distant metastasis [2].

The current standard treatment for MPT is surgical excision of the tumour with sufficient resection margins. The addition of postsurgical radiation therapy did not result in substantial improvement in overall survival, and the benefit of adjuvant systemic therapy has not been tested in randomised clinical trials [3, 4]. Owing to the rare nature of the neoplasm and our incomplete understanding of the molecular characteristics of the disease, there are no effective systemic or targeted treatment options for MPT despite high rates of recurrence.

Recent studies have improved our understanding of the molecular characteristics of MPT [4, 5]. Scientists have identified recurrent chromosomal alterations [6, 7], gene expression characteristics [7, 8], and somatic mutations [9, 10]. However, many have primarily focused on genomic profiling of a wide range of phyllodes neoplasms, and thus included large proportions of benign and borderline phyllodes tumours. Furthermore, few studies have addressed the potential in vivo application of targeted therapies in MPT, owing to the lack of available cell lines or animal models.

Here, we presented the genomic and transcriptomic characteristics of MPT using various tumour tissues and suggested gene expression-based molecular subtypes of MPT. Additionally, we elucidated the driver and microenvironment relationship in this biphasic neoplasm using a patient-derived xenograft (PDX) model derived from MPT. Finally, we proposed novel therapeutic approaches for MPT by targeting the platelet-derived growth factor receptor (PDGFR) and phosphoinositide 3-kinase (PI3K)/mammalian target of rapamycin (mTOR) pathways.

## Methods

### Patients and biospecimens

Frozen tumour tissues of MPTs were obtained from the Institutional Review Board (IRB)-approved breast cancer biospecimen repository at Seoul National University Hospital (IRB no. 1509-032-702). Tissues were obtained from surgical specimens during curative surgeries. Matched germline DNA was obtained from the peripheral blood of the patient. Formalin-fixed, paraffin-embedded (FFPE) MPT tissues were obtained from the Department of Pathology, Seoul National University Hospital (IRB no. 1402-054-555).

### Patient-derived xenograft models

To develop the PDX models, surgically resected tumour tissues were minced into approximately 2 mm pieces and transplanted into the fourth mammary fat pad of 8-week-old female NOD/SCID/IL-2γ-receptor null (NSG, Jackson Lab, USA) mice. When the MPT PDX tumour exceeded 500 mm^3^ after transplantation into the mouse mammary fat pad, it was excised and cut into 2–3 mm^3^ pieces. Subsequently, a hole was made in the mammary fat pad of the mouse to be transplanted, and a piece of PDX tumour was placed in the hole. Thus, MPT PDX models were established in several passages (P1 and P2). Tumour volumes and body weight of mice were measured once or twice a week.

### Whole-exome sequencing (WES) and data processing

Exome libraries captured using the SureSelect Human All Exon V4 or V5 kit (Agilent, Santa Clara, CA, USA) for WES were sequenced on an Illumina platform. Most bioinformatic analyses of sequencing data were performed using the computing server at the Genomic Medicine Institute Research Service Center. We aligned the DNA sequence reads to the human reference genome (GRCh37) or a combined reference genome of human (GRCh37) and mouse (mm10) using Burrows–Wheeler Aligner (BWA) [11]. Sorting of reads and marking of PCR-duplicated reads were performed using Picard (http://broadinstitute.github.io/picard/). For samples using a combined reference genome of human and mouse, we removed mouse sequence reads according to additional processing procedures [12]. Thereafter, we performed preprocessing procedures for bam files, including local realignment around insertions/deletions (indels) and base recalibration, according to the Genome Analysis Toolkit (GATK) best practices document [13, 14].

### Mutational landscape analysis using WES data

We discovered somatic mutations using MuTect [15] for single-nucleotide variants (SNVs) and GATK IndelGenotyper V2 [14] for indels. ANNOVAR [16] was used to annotate the somatic mutations. We used the following criteria to identify true positive variants in the exome data: alt allele depth ≥4 and “MuTect=PASS”. For indels, total depth ≥8, alt allele depth ≥6, and alt allele frequency ≥0.1. Only coding or splice-site variants were maintained.

The number of variants that passed the above criteria was described as the number of “Total” variants in Supplementary Table S1. Somatic mutation burden was calculated by the number of “Total” variants per megabase for exome. We performed an analysis to identify mutational processes using deconstructSigs [17] based on mutational signatures in COSMIC [18] with default settings or with additional normalisation. SigMA can be used to detect mutational signatures in sequencing data with a small number of mutations [19]. We used this tool to more accurately detect signature 3, which is frequently found in breast cancer, using a web-based interface (http://compbio.med.harvard.edu/sigma/). As it is difficult to filter germline mutations in one sample without a matched normal sample, the “mpt-06” sample was excluded from the somatic mutation burden and mutational signature analysis.

Additional filters were applied to identify more noteworthy variants. Following removal of variants annotated as “synonymous” or “unknown” by ANNOVAR [16], we only retained variants that rarely exhibited or did not report in the general population database, including the 1000 Genomes Project, Exome Aggregation Consortium, and Exome Sequencing Project. The number of variants that passed the additional filter was described as the number of “Noteworthy” variants in Supplementary Table S1. To identify somatic mutations in one tumour sample without a matched blood sample (mpt-06), the variants considered germline variants by the Korean germline variant database [20] were removed from the variants identified using the MuTect tumour-only mode.

MutationMapper [21, 22] was used for lollipop plot visualisation of specific mutations. Additional interpretation of mutations was performed by MutationMapper and Oncotator [23]. Cancer-related genes were defined on the basis of the COSMIC Cancer Gene Census [24]. We surveyed clinically relevant variants to discover potential therapeutic targets based on the TARGET database v3 from the Broad Institute (https://software.broadinstitute.org/cancer/cga/target).

### Somatic copy number alterations (SCNAs) analysis

We discovered SCNAs from exome sequencing data using EXCAVATOR [25], the R package DNAcopy, and CNVkit [26]. After generation of read coverage for all samples using CoNIFER [27], we calculated the log2 ratio of tumour and matched blood samples, and used these values as input for DNAcopy analysis. For one sample without a matched blood sample, we utilised a pool of blood samples that used the same exome capture kit as the baseline for DNAcopy and CNVkit analysis. The SCNAs identified from the DNAcopy are shown in Supplementary Fig. S2a. To identify important SCNAs, we detected genes in regions with copy numbers >4 or genes where mutations appeared among cancer-related genes, or recurrently variable regions from results by EXCAVATOR.

### RNA sequencing (RNA-seq) and data processing

Library construction for RNA-seq was performed using the TruSeq RNA Sample Prep Kit v2 for most samples, TruSeq RNA Access Library Prep kit for FFPE, or TruSeq Stranded mRNA Prep kit (Illumina, San Diego, CA, USA) for several MPT PDX samples. Sequencing of cDNA libraries was performed using the Illumina platform. We used a human genome reference and a combined reference for human and mouse to align sequence reads by spliced transcripts alignment to a reference (STAR) aligner [28]. The following procedures were conducted referring to the best practices workflow for RNA-seq of GATK. The number of reads for each Ensembl annotated gene was quantified from pre-processed data using HTSeq-count [29]. We applied parameters that were considered appropriate for each sequencing data. Raw read counts from HTSeq-count were converted to fragments per kilobase of exon per million reads mapped (FPKM) values using the rpkm() function of the R package edgeR [30].

To find *MED12* mutations from RNA-seq data of FFPE, we manually examined only known mutation sites in *MED12* exon 2, previously found in phyllodes tumour [10] from the bam files using the Integrative Genomics Viewer (IGV).

### Differential gene expression analysis

Differentially expressed genes (DEGs) were identified using the R package DESeq2 [31]. We only retained genes with an adjusted *p* value < 0.05, log2 fold-change ≥1, and average of expression (FPKM) across all samples ≥1 to identify more important DEGs from the results of differential gene expression analysis. To identify noteworthy enriched pathways from DEGs, we used the DAVID Bioinformatics Database functional annotation tool (http://david.abcc.ncifcrf.gov/). Statistical significance was set at *p* < 0.05. The ClueGO [32] Cytoscape plugin was used to visualise the enriched Kyoto Encyclopedia of Genes and Genomes (KEGG) pathways [33]. Significance in ClueGO was determined by the p-value corrected with Bonferroni step-down.

### Visualisation of transcriptome profiles

After adding 1 to the FPKM of each gene, these values were log transformed and mean-centred. The adjusted expression values were hierarchically clustered based on uncentered correlation and average linkage by cluster 3.0 [34]. Visualisation of the heatmap was performed using Java Treeview [35].

Read counts of each gene from HTSeq-count were transformed by the variance stabilising transformation (vst) function using the R package DESeq2 [31]. After determining the variance for each gene, the 1000 most variable genes were used for principal component analysis (PCA). We generated a PCA plot based on the first two principal components (PC1 and PC2) using R package’s ggplot2 or Prism (GraphPad).

### Molecular classification of MPT samples

To identify classifier genes which divide MPT samples into the “Epithelial” and “Fibrous” subtypes, we applied the criteria below and selected 32 genes among noteworthy DEGs identified in differential gene expression analysis between the two subtypes of nine MPT fresh frozen (FF) tissues: (1) protein-coding gene, (2) log2 of fold-change among genes overexpressed in “Epithelial” ≥8, log2 of fold-change among genes overexpressed in “Fibrous” ≥2, (3) minimum difference of expression (FPKM) between the two groups of MPT FF tissues ≥1, and (4) mean expression (FPKM) in 28 MPT FFPE tissues ≥2.

### Histological analysis

MPT PDX tumours were resected once the tumour volume reached 200, 500, and 1000 mm^3^, and paraffin-embedded blocks were prepared and mounted on microscope slides. To quantify the ratio of epithelial cells in these MPT PDX tumours, an image analysis program “QuPath” was used. To discriminate between human mesenchymal cells and mouse epithelial cells histologically, PDX tissue slides were rehydrated, and immunostaining was performed with anti-human HLA class 1 (Abcam, 1:800). For human and mouse pan-centromeric FISH followed by anti-FITC staining, we used Star*FISH human and mouse pan-centromeric probes-FITC (Cambio) according to the manufacturer’s protocol. Briefly, after rehydration, slides were incubated with pepsin solution and quenched with glycine solution. Following washing with PBS, slides were post-fixed in paraformaldehyde solution. Thereafter, tissue slides were dehydrated, stained with chromosome paint overnight, and subsequently washed with formamide, stringency, detergent wash solution, and PBS sequentially, followed by HRP-conjugated anti-FITC (Abcam, 1:50) immunostaining for 1 h at room temperature. After mounting, slides were observed under a light microscope.

### Single-cell RNA sequencing (scRNA-seq) data processing and analysis

Raw 3′ scRNA-seq data were aligned using Cell Ranger [36] to generate a count matrix. Sequencing reads were aligned to the human mouse (GRCh38-mm10) chimeric reference genome. For the analysis of tumour cells treated with imatinib, PKI-587, and vehicle, we removed doublets in each group using DoubletFinder [37]. SoupX [38] was used to remove technical ambient RNA. Expression matrices were further processed in R using Seurat [39]. The “Subset” function was applied to separate mouse and human cells. After removing unwanted cells, the “NormalizeData” function with the “LogNormalize” method was used to normalise filtered gene-barcode matrices, and the top 2,000 features were found using the “FindVariableFeatures” function with the “vst” method. Scaling was performed on the gene expression matrices using the “ScaleData” function. Thereafter, we performed PCA on scaled data and used the first 30 principal components (PCs) for further analysis. Harmony was used to integrate the data. The top 30 PCs were used as input to the UMAP dimension reduction. Nearest-neighbour graphs were calculated using “FindNeighbors” with the top 30 PCs and “FindClusters” function applied with a resolution of mouse = 0.8 and human = 0.8, respectively. Harmony [40] was run on the PCA matrix using the default parameters with a library batch. Human cells containing at least 1% mouse reads were removed, and filtered human cells were considered the tumour cell population. The Seurat “AddModuleScore” was used to calculate the PI3K pathway score. Gene sets with *PIK3CA*, *PIK3CB*, *AKT1*, *MTOR*, and *RPS6KB1* were used to calculate module scores.

### Human and mouse cells separation in MPT PDX

To separate human and mouse cells from MPT PDX tumours, we used the MACS mouse cell depletion kit (Miltenyi Biotec) designed for isolating human cell populations in xenograft tumours using magnetically labelled beads targeting mouse cells. To dissociate the MPT PDX tumour, we resected the PDX tumour at 1000 mm^3^ and dissociated it using a tumour dissociation kit with a gentle MACS dissociator (Miltenyi Biotec) according to the manufacturer’s protocol. Dissociated cells were labelled with mouse-specific magnetic beads and separated using LS columns to obtain unlabelled flowing human cells. Following human cell isolation, bead-labelled mouse cells were detached from the LS column.

To verify whether cells were successfully separated by the host species, RNA was extracted from cells collected from each human and mouse using TRIzol reagent. Using a cDNA synthesis kit (Takara), cDNA was synthesised, and qRT-PCR was performed using the human GAPDH primer (F:5’-GAG TCC ACT GGC GTC TTC-3’ R:5’-GGA GGC ATT GCT GAT GAT C-3’) and mouse GAPDH primer (F:5’-GCC TTC CGT GTT CCT ACC-3’ R:5’-GCC TGC TTC ACC ACC TTC-3’).

### Drug efficacy test in the MPT PDX

Drug treatment was initiated after the tumours reached approximately 200 mm^3^. Mice were randomly divided into three treatment groups consisting of five mice in each group: (1) vehicle (0.5% methylcellulose, daily per oral), (2) imatinib mesylate (100 mg/kg, 0.5% methylcellulose, daily per oral), and (3) PKI-587 (25 mg/kg, dissolved in 5% dextrose, 0.3% lactic acid, weekly intravenous injection) for 3 weeks. The tumour volume and body weight of the mice were measured once or twice a week. Volume was calculated as (length × width^2^)/2.

### Western blotting

For protein extraction, a piece of tumour tissue (approximately 1 mm^3^) was homogenised in ice-cold M-PER containing a protease and phosphatase inhibitor cocktail (Thermo Scientific) using a disposable homogeniser (BioMasher II tissue homogeniser, Nippi), briefly sonicated, and centrifuged at 14,000 × *g* for 20 min at 4 °C. Total proteins were separated by sodium dodecyl sulfate-polyacrylamide gel electrophoresis and transferred onto a polyvinylidene difluoride membrane. Following blocking, membranes were probed with specific antibodies against phospho-Akt (Ser473), Akt, phospho-mTOR (Ser2448), mTOR, phospho-MAPK (Thr202/Tyr204), MAPK, phospho-PDGFRa (Tyr849)/PDGFRb (Tyr857), PDGFRb (Cell Signaling Technology), and β-actin (Santa Cruz Biotechnology), followed by treatment with horseradish peroxidase-conjugated secondary antibodies. Target proteins were detected using Amersham Imager 600 (GE Healthcare) developed with an enhanced chemiluminescent substrate (Thermo Scientific).

### Utilisation of in-house data

We utilised WES data on 18 invasive ductal carcinoma (IDC) samples and transcriptome data from 32 non-tumour breast tissues, 41 IDC tissues, 46 PDX tissues derived from IDC, and 6 normal mouse fat pads. The sequencing data of samples were generated similar to the samples used in this study for a separate project, to characterise genomic profiles of primary breast tumour and PDX tumour tissues, which will be presented separately (IRB no. 1402-054-555).

### Statistical analyses

Most statistical calculations were performed using Prism version 8.0 for Windows (GraphPad software, La Jolla, CA, USA, www.graphpad.com). To identify statistically significant differences, the Mann–Whitney test was used for most statistical analyses. Fisher’s exact test was used for comparison of the frequency of signature 3 between MPT and IDC and the frequency of *MED12* mutation according to molecular subtype. The chi-square test was used for comparison of degree of stromal overgrowth according to the molecular subtype and comparison of cellular composition upon drug treatment. The Kruskal–Wallis test was used for comparison of expression level of cell-type markers based on mouse transcriptome from MPT, IDC PDX, and normal fat pads. The Wilcoxon test was used for tumour growth comparison of drug-treated “MX-99” xenograft model. The Student’s *t* test was used for comparison of PI3K activity upon drug treatment. Survival curves were created using the Kaplan–Meier method and compared using the log-rank test. All data are presented as the mean ± standard deviation (SD). *p* values < 0.05 were considered significant and indicated by *p < 0.05, **p < 0.01, ***p < 0.001, ****p < 0.0001.

## Results

### Genomic and transcriptomic characteristics of MPT of the breast

**Fig. 1:**
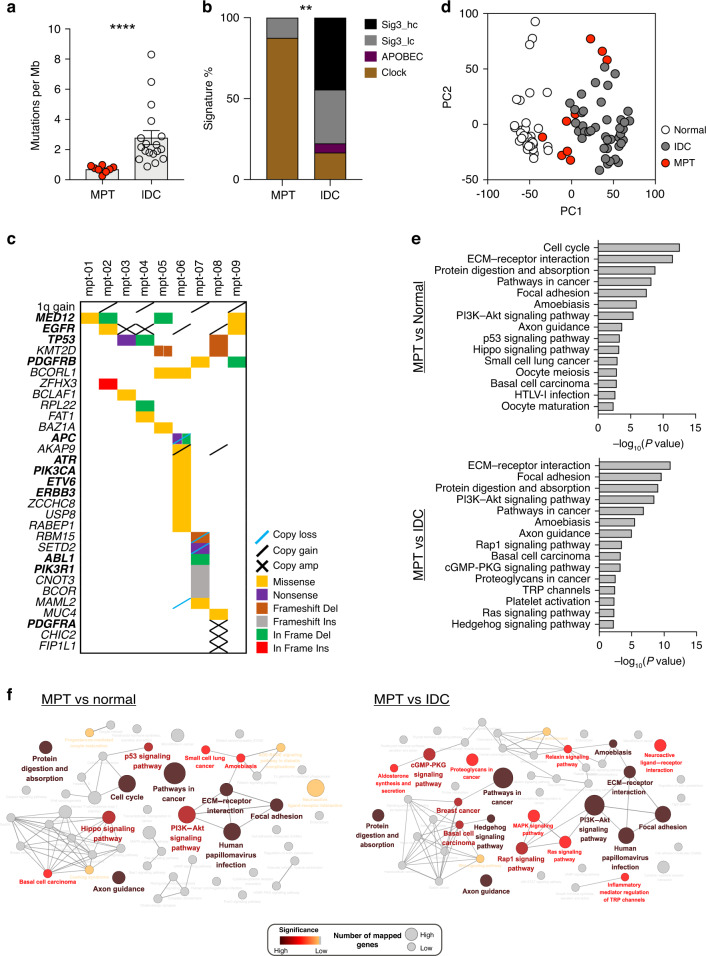
Unique molecular characteristics of malignant phyllodes tumours (MPTs). **a** Prevalence of somatic mutations, measured by the number of somatic mutations per Mb of covered target sequence, in MPT compared to that in invasive ductal carcinoma (IDC) (MPT, *n* = 8; IDC, *n* = 18; Mann–Whitney test). **b** Comparison of the prevalence of signatures between MPT and IDC identified by SigMA (MPT, *n* = 8; IDC, *n* = 18; Fisher’s exact test). “Sig3 hc” means signature 3 with high confidence, and “Sig3 lc” means signature 3 with low confidence. **c** Somatic mutations and copy number alterations in cancer-related genes and specific regions in the nine MPT. Genes associated with clinical action are denoted in bold font, based on TARGET v3 from the Broad Institute. Genes marked with “copy amp” indicate genes with a copy number ≥5. **d** PCA plot showing unique gene expression profiles of MPT compared to normal breast and IDC tissues. **e** KEGG pathways enriched with upregulated genes in MPT compared to normal breast tissues (top) or IDC tissues (bottom). **f** Cytoscape network analysis showing significantly altered pathways in MPT compared to normal breast tissues (left) or IDC tissues (right). The colour-filing nodes reflect statistical significance of the term (pathway). Node size refers to the number of genes associated with the term among the differentially expressed genes. Terms with high significance were clearly labelled. **p < 0.01, ****p < 0.0001.

We obtained fresh frozen (FF) tissues from nine MPT patients in our biorepository and performed exome and transcriptome sequencing (Supplementary Table S1). Compared to exome sequencing data generated from an independent cohort of 18 IDC tissues, the MPT showed a significantly lower incidence of somatic mutations (0.7/Mb for MPT and 2.79/Mb for IDC) (Fig. 1a and Supplementary Fig. S1a). The most prevalent somatic mutation signature, as proposed by Alexandrov et al. [41], of MPT was the C ∙ G → T ∙ A substitutions resulting from endogenous DNA damage (Supplementary Fig. S1b–d). The low incidence of the *BRCA*-related mutational signature (signature 3) and APOBEC editing-related signatures (signatures 2 and 13) suggested that the mutational process of MPT is distinct from that of IDC of the breast [42] (Fig. 1b and Supplementary Fig. S1d). The incidence of structural variations and somatic mutations in cancer-related genes in nine MPT is shown in Fig. 1c (Supplementary Data 1). Consistent with previous reports [4, 9, 43–45], we observed a high rate of *MED12* mutations in our series (4/9, 44.4%). Additionally, somatic mutations in *PIK3CA*, *PIK3R1*, *PDGFRB, SETD2*, and *TP53* were observed among the nine MPT. Regarding structural variations, we observed heterogeneous structural alterations, including 1q gain in four cases (44.4%) and EGFR amplifications in four cases (44.4%) (Fig. 1c and Supplementary Fig. S2a).

The transcriptome sequencing data of the nine MPT were compared with 32 normal breast tissues and 41 IDC tissues to determine the gene expression characteristics of the MPT. PCA using gene expression profiles revealed that MPT have unique characteristics compared to IDC or normal breast tissues (Fig. 1d). Pathway enrichment analysis using DEGs showed that, in addition to the cell cycle and pathways in cancer, genes involved in extracellular matrix (ECM) interactions and the PI3K signalling pathway were upregulated in MPT compared to normal breast tissues. The genes involved in ECM interactions and PI3K signalling were also upregulated in MPT compared to IDC (Fig. 1e). Alternatively, when compared to normal breast tissues, gene sets related to metabolism and the PPAR signalling pathway were downregulated in MPT, and when compared to IDC, genes involved in cell adhesion molecules and tight junctions were downregulated in MPT (Supplementary Fig. S2b). Interaction network analysis using Cytoscape [46] further revealed that ECM-related pathways and PI3K-Akt signalling pathways were significantly enriched in MPT (Fig. 1f). These findings suggested that MPT has unique genomic and transcriptomic characteristics that differ from those of the IDC of the breast.

### Epithelial and mesenchymal features determine the clinically-relevant molecular subtypes of MPT

**Fig. 2:**
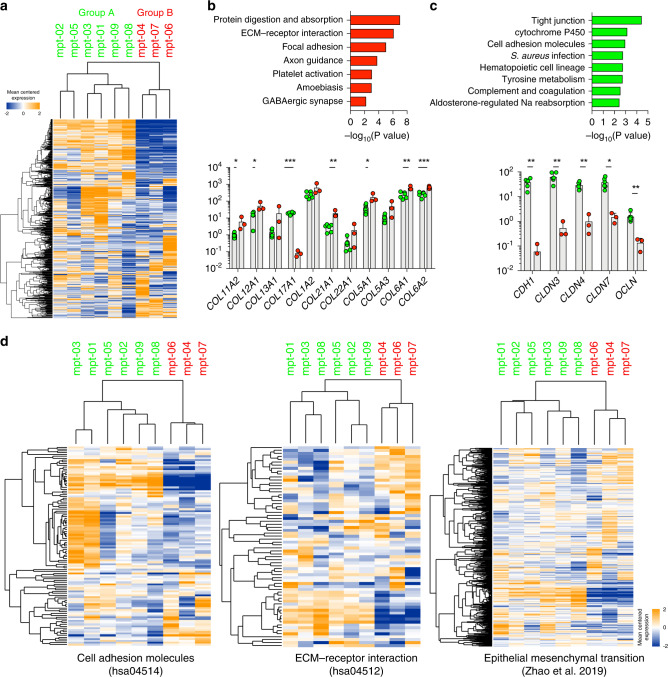
Two molecular subtypes of malignant phyllodes tumours (MPTs). **a** Unsupervised clustering of nine MPT using expression levels of the top 2000 variable genes except for genes with low expression levels. **b** Significant pathways enriched with upregulated genes in “Group B” (top) and mRNA expression levels (FPKM) of various collagens (bottom) (Mann–Whitney test). **c** Significant pathways enriched with upregulated genes in “Group A” (top) and mRNA expression levels (FPKM) of epithelial cell markers such as cadherin, claudins, and occludin (bottom) (Mann–Whitney test). **d** Heatmaps showing the clustering of subtypes based on genes involved in cell adhesion molecules, ECM–receptor interactions, and EMT. *p < 0.05, **p < 0.01, ***p < 0.001.

Unsupervised clustering using gene expression data suggested that MPT can be classified into two distinct molecular subtypes (Fig. 2a and Supplementary Fig. S3a). We identified DEGs between the two molecular subtypes of MPT (Supplementary Data 2 and Supplementary Fig. S3b). One subtype was enriched with genes involved in protein digestion and absorption, ECM-receptor interaction, focal adhesions, and axon guidance (Fig. 2b). Many mesenchyme-related genes, such as various types of collagens [47], were significantly upregulated (Fig. 2b). The other subtype showed upregulation of genes involved in tight junctions, cell metabolism, cell adhesion molecules, and various immune-related processes (Fig. 2c). This subtype showed upregulation of epithelial markers such as *CDH1*, *CLDN3*, *CLDN4*, *CLDN7*, and *OCLN* (Fig. 2c) [48–50]. In addition to examining expression levels of individual genes, we determined global expression patterns of genes associated with cell adhesion molecules (KEGG ID: hsa04514), ECM–receptor interaction (KEGG ID: hsa04512) from the KEGG database (http://www.genome.jp/kegg/) [33], and the epithelial-to-mesenchymal transition (EMT) [51], as the above gene sets could represent the epithelial or stromal spectrum of the MPT. Unsupervised clustering based on expression profiles of genes in the three gene sets resulted in the same classification of the nine tumours (Fig. 2d). These findings indicated that human MPT can be classified into two distinct molecular subtypes based on their mesenchymal and epithelial gene expression characteristics. We named the observed subtypes as “Epithelial” and “Fibrous”, respectively which reflect the biphasic nature of fibroepithelial neoplasms.

**Fig. 3:**
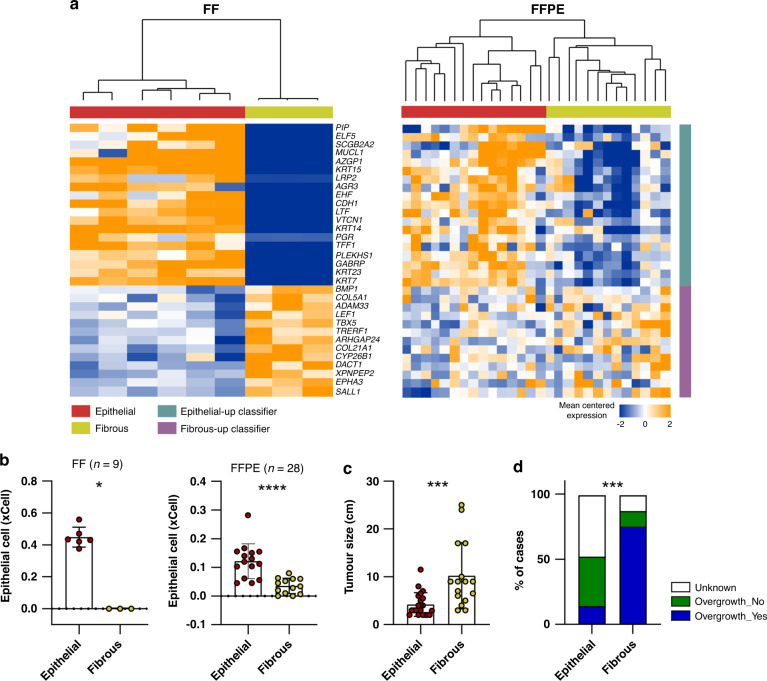
Clinical implications of molecular subtypes of malignant phyllodes tumours (MPTs). **a** Hierarchical clustering of MPT FF (left) or FFPE (right) samples using the expression levels of the 32-classifier genes. **b** Comparison of the xCell scores of epithelial cells between the two groups in MPT FF (left) or FFPE (right) tissues (Mann–Whitney test). **c** Comparison of tumour size according to the molecular subtypes in MPT FF and FFPE tissues (“Epithelial”, *n* = 21; “Fibrous”, *n* = 16; Mann–Whitney test). **d** Comparison of degree of stromal overgrowth according to the molecular subtypes in MPT FF and FFPE tissues (“Epithelial”, *n* = 21; “Fibrous”, *n* = 16; Chi-square test). *p < 0.05, ***p < 0.001, ****p < 0.0001.

We identified that mutations in *MED12* were found only in the “Epithelial” subtype (4/6 for “Epithelial,” 0/3 for “Fibrous”; Fisher’s exact test, *p* = 0.1667; Fig. 1c). Notably, MPT samples belonging to the “Fibrous” subtype showed higher expression levels of *PDGFRB* compared to those of MPT samples belonging to the “Epithelial” subtype, although the difference was not significant (Supplementary Fig. S3c). As it is known that the expression level of *PDGFRB* increases with increasing grade and that the frequency of *MED12* mutation decreases with increasing grade in the spectrum from benign to malignant phyllodes tumour [44, 52], we speculated that “Fibrous” would be a more advanced group compared to “Epithelial” within the malignant histologic category of phyllodes tumours. We performed RNA-seq using additional 28 MPT FFPE tissues to investigate the impact of gene expression features of molecular subtypes of MPT on clinical parameters of patients with MPT. We developed a gene expression signature consisting of 32 genes that could be used to classify MPT samples into two distinct groups and applied it to classify MPT FFPE samples (Supplementary Data 2 and Fig. 3a). We confirmed that the MPT FFPE samples were classified into two clusters based on the 32-gene expression signature (Fig. 3a). MPT FFPE samples also tended to form clusters in the PCA plot according to the group classified by the 32-gene expression signature (Supplementary Fig. S3d). To determine the degree of epithelial gene expression in tumours in each subgroup, we used xCell [53], a gene signature-based analytical tool that has been developed to identify specific cellular types from bulk RNA-seq data sets. As shown in Fig. 3b, xCell-based epithelial gene expression was significantly associated with molecular subtypes in MPT FF and FFPE tissues. The subgroup enriched with mesenchymal-related pathways showed significantly lower levels of xCell-based epithelial genes (Fig. 3b). We confirmed that mutations in *MED12* exon 2, which are frequently mutated in phyllodes tumours, including mutations in codon 44 (p.G44) of *MED12*, appear only in the “Epithelial” group in FFPE tissues as in FF tissues (3/15 for “Epithelial,” 0/13 for “Fibrous”; Fisher’s exact test, *p* = 0.2262; Supplementary Fig. S3e). Thus, we propose that the presence of the *MED12* mutation is associated with the molecular subtype of MPT (7/21 for “Epithelial,” 0/16 for “Fibrous”; Fisher’s exact test, *p* = 0.0124). The patients with “Epithelial” tumours showed significantly smaller tumour size compared to that of the patients with “Fibrous” tumours (Fig. 3c). These molecular subtypes were also significantly associated with the degree of stromal overgrowth, another well-known prognostic factor for MPT [54] (Fig. 3d). Although these molecular subtypes were markedly associated with histologic stromal overgrowth patterns, the histologic and molecular classifications showed similar metastasis patterns in both groups (Supplementary Fig. S3f and Supplementary Table S2).

### Cancerous mesenchymal component and microenvironmental epithelial component of MPT

**Fig. 4:**
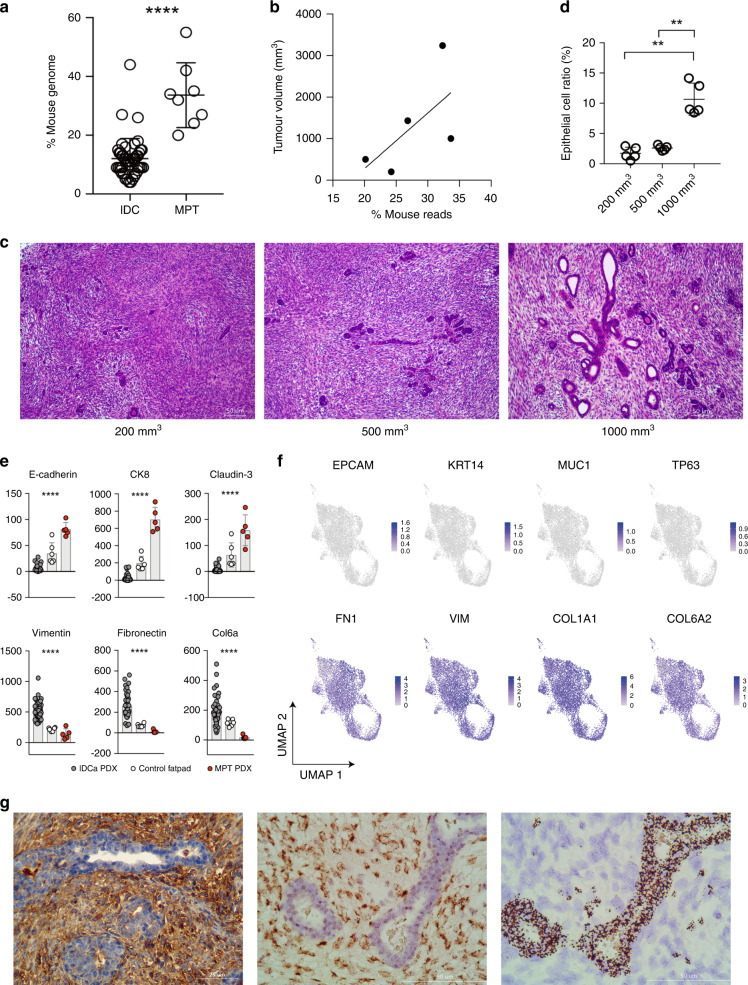
Role of epithelial cells and stromal cells in malignant phyllodes tumours (MPTs). **a** Proportion of RNA-seq reads originating from murine cells in PDX derived from invasive ductal carcinoma (IDC) and MPT (Mann–Whitney test). **b** Correlation between MPT tumour volume and the proportion of RNA-seq reads originating from murine cells in the MPT PDX**. c** Haematoxylin and eosin staining results of MPT PDX tumour resected at volumes 200 mm^3^ (left), 500 mm^3^ (middle), and 1000 mm^3^ (right). **d** Proportion of epithelial cells according to tumour volume (Mann–Whitney test). **e** Expression levels of epithelial cell markers (top line) and mesenchymal stromal cell markers (bottom line) based on mouse transcriptome in normal mouse fat pads and PDX derived from MPT and IDC (Kruskal–Wallis test). **f** UMAP embeddings of epithelial markers expressing grafted human tumour cells (top) and fibroblast markers expressing grafted human tumour cells (bottom) (*n* = 8105). **g** Identification of human mesenchymal cells and mouse epithelial cells by anti-human HLA class 1 staining (left), anti-FITC staining after human-specific centromeric FISH (middle), and mouse-specific centromeric FISH (right). **p < 0.01, ****p < 0.0001.

Among the nine MPT tumours used above, we obtained fresh tumour tissue from one patient (“mpt-07”) during the curative surgery and develop a PDX model using NSG mice (“MX-99”). Previous studies have shown that murine cells replace the microenvironmental cells around human tumours after patient-derived tumours are engrafted into mice [55, 56]. Accordingly, we discriminated between the transcriptome of the tumour and the microenvironment using species-specific genome sequences [12, 57]. Notably, when compared to those of PDX tumours derived from IDC, which are of epithelial origin, the RNA-seq data of PDX tumours derived from MPT tumours had a significantly higher proportion of mouse mRNA reads (Fig. 4a). Furthermore, when MPT PDX tumours were transplanted into multiple NSG mice and tumour tissues were collected at various tumour sizes, we observed that the proportion of mouse mRNA reads increased along with tumour size (Fig. 4b). These findings indicated that murine cells within the mouse microenvironment increase in number as the tumour grows. Histological examination of the MPT PDX tumours at different tumour sizes revealed that the mesenchymal component constitutes most of the tumour area during the early period, while the epithelial components gradually increased as the tumours grew (Fig. 4c, d).

The mouse reads obtained from MPT PDX tumours showed distinct gene expression patterns compared to those of normal mouse mammary fat pads, including upregulation of the cell cycle-related gene sets (Supplementary Fig. S4). To determine whether the epithelial-like morphology of separated murine cells was supported by gene expression profiles, we compared the expression levels of well-known epithelial and mesenchymal genes. When compared with the mouse reads from PDX tumours derived from IDC and normal mouse fat pads, mouse reads from the MPT PDX tumours showed upregulation of epithelial markers and downregulation of mesenchymal markers (Fig. 4e).

Thereafter, we performed scRNA-seq of the MPT PDX to confirm the identity of human MPT cells at single-cell resolution (Supplementary Fig. S5a). As shown in Fig. 4f, the expression of epithelial genes was low, whereas the expression of fibroblast genes was high in the cancerous human cells of the MPT PDX. We further isolated human and mouse cells from MPT PDX tumour tissues using the magnetic cell separation technique (Supplementary Fig. S5b) [58]. The isolated human tumour cells displayed a spindle-shaped, fibroblast-like appearance, while murine cells formed adherent structures similar to epithelial cells (Supplementary Fig S5c). To determine the origin of the cells more directly, we stained the MPT PDX tumour with a human-specific antibody and observed that only mesenchymal cells were positively stained with an anti-human HLA class 1 antibody (Fig. 4g). Furthermore, the epithelial cells stained positive for mouse-specific centromeric probes, while mesenchymal cells stained positive for human-specific probes by the species-specific FISH experiment [59] (Fig. 4g and Supplementary Fig. S5d). In addition, we developed a PDX model (“PDX78”) using tissues obtained from another MPT patient and performed bulk RNA-seq analysis. Unlike the previous MPT PDX (“MX-99”), we did not find conclusive evidence showing an increase of the murine microenvironmental component in this MPT PDX (“PDX78”) (Supplementary Fig. S5e). However, we confirmed that the expression of fibroblast genes, not epithelial genes, was also enriched in cancerous human cells of the “PDX78” as in the “MX-99” (Supplementary Fig. S5f).

Collectively, our results indicated that the mesenchymal cells of the MPT, and not the epithelial cells, have the capacity to survive and form PDX tumours within the mouse microenvironment. Furthermore, the murine epithelial component increased as it formed glandular structures within PDX tumours. These data suggested that, unlike carcinomas of epithelial origin, MPT consist of cancerous cells that have fibroblast-like features and a tumour microenvironment with proliferating epithelial cells.

### Targeting mesenchymal component provides potential therapeutic approaches in MPT

**Fig. 5:**
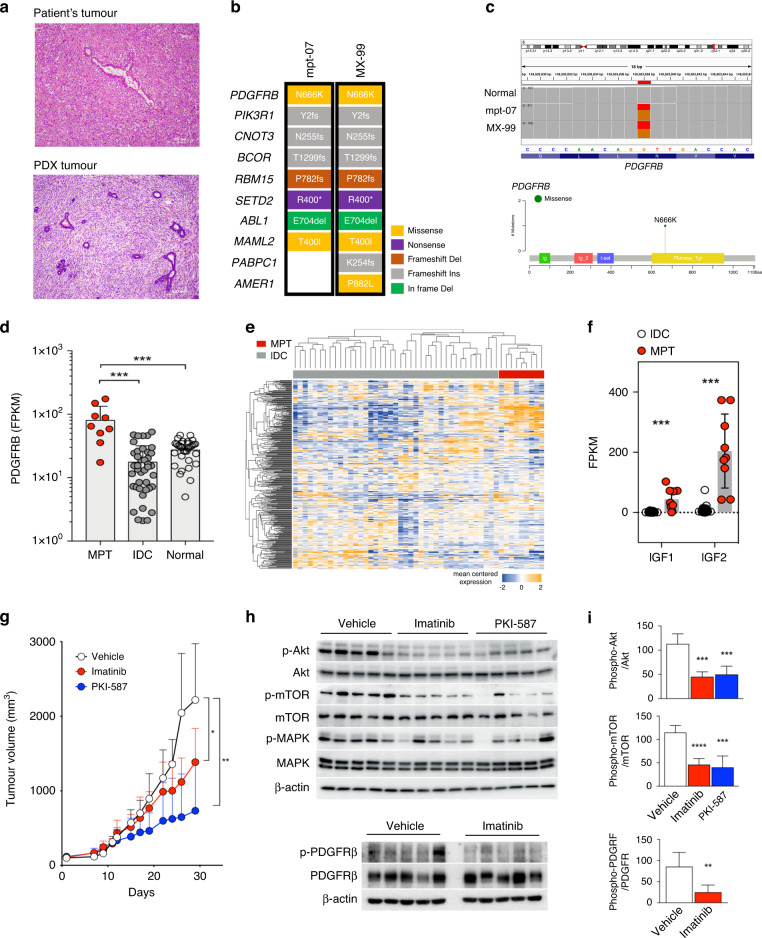
Discovery of potential therapeutic target of malignant phyllodes tumours (MPTs). **a** Hematoxylin and eosin stained picture of primary tumour “mpt-07” of the patient with MPT (top) and MPT PDX tumour “MX-99” (bottom). **b** Somatic mutations in cancer-related genes in primary tumours (mpt-07) and matched PDX tumours (MX-99). **c** Visualisation in Integrative Genomics Viewer (IGV) shows p.N666K somatic mutation of *PDGFRB* in the primary and PDX tumours (top), and the lollipop plot shows the p.N666K mutation in the *PDGFRB* kinase domain (bottom). **d** *PDGFRB* mRNA expression levels in normal breast tissues, invasive ductal carcinoma (IDC) tissues, and MPT (Mann–Whitney test). **e** Heatmap showing the expression patterns of genes involved in the PI3K/Akt/mTOR pathway in MPT and IDC. Genes with an average FPKM across all samples ≥1 were selected among genes in the PI3K-Akt signalling pathway (KEGG ID: hsa04151) and mTOR signalling pathway (KEGG ID: hsa04150). **f** mRNA expression levels of *IGF1* and *IGF2* in MPT and IDC (Mann–Whitney test). **g** Tumour growth in the “MX-99” xenograft model treated by vehicle, imatinib, and PKI-587 (Wilcoxon test). **h** Western blot analysis of downstream signalling pathway molecules in xenograft tumours treated with vehicle, imatinib, and PKI-587. **i** Quantitative analysis of western blot results (Mann–Whitney test). *p < 0.05, **p < 0.01, ***p < 0.001, ****p < 0.0001.

Microscopic examination showed that the PDX tumour reproduced the histologic features of the patient’s original tumour (Fig. 5a). The PDX tumour showed genomic and transcriptomic profiles similar to those of the primary tumour (Fig. 5b and Supplementary Fig. S6). The primary and PDX tumours had somatic mutations in *PDGFRB* (NM_002609: p.N666K) (Fig. 5b, c). *PDGFRB* N666K has been reported as an oncogenic mutation in rare mesenchymal disorders, infantile myofibromatosis, and cells that harbour the mutation respond to imatinib mesylate [60]. Recently, this mutation was also found in phyllodes tumours and was reported to respond to pazopanib, which is primarily used for soft tissue sarcomas [61]. *PDGFRB* is typically expressed in mesenchymal cells and not in epithelial cells [62]. Examination of RNA-seq data further revealed that when compared to IDC or normal breast tissues, *PDGFRB* mRNA expression was significantly upregulated in MPT samples, suggesting that *PDGFRB* levels may correlate with the degree of mesenchymal features (Fig. 5d and Supplementary Fig. S3c).

The “MX-99” tumour also harboured somatic mutation in *PIK3R1* (NM_181504: p.Y2fs), which is a regulatory subunit for PIK3CA with tumour-suppressor properties [63]. Somatic mutations in *PIK3R1* are often associated with increased PI3K/Akt signalling in cancer [64]. Our data also showed that MPT tissues had significant dysregulation of genes involved in the PI3K/Akt/mTOR pathway compared with IDC tissues (Fig. 5e). Additionally, *IGF1* and *IGF2*, which activate PI3K signalling in tumours in an autocrine manner, were also significantly upregulated in MPT tumours compared to IDC (Fig. 5f).

To test whether the genetic characteristics of the PDX model can be exploited to develop therapeutic approaches, we treated the PDX model with imatinib mesylate and PKI-587 [65] to target the PDGFR and PI3K/mTOR pathways, respectively. Both targeted agents significantly suppressed in vivo tumour growth of MPT in the PDX models, with higher efficacy for PKI-587 (Fig. 5g). Treatment with PKI-587 and imatinib mesylate effectively inhibited the phosphorylation of Akt and mTOR in PDX tumours, and imatinib inhibited PDGFRB phosphorylation (Fig. 5h, i). We additionally performed scRNA-seq analysis of drug-treated PDXs to identify changes in tumour cells upon drug treatment. Before comparative analysis between vehicle- and drug-treated PDXs, we first confirmed that human tumour cells in vehicle- and drug-treated MPT PDX had high expression of fibroblast genes, which is consistent with previous findings (Supplementary Fig. S7a). Through comparative analysis, we observed a decrease in the activity of the PI3K pathway and a decrease in the proportion of proliferating cells in the tumour cell population in drug-treated MPT PDX when compared with vehicle-treated MPT PDX (Supplementary Fig. S7b, c). These data demonstrated the potential efficacy of targeted approaches for MPT treatment.

## Discussion

In the present study, we characterised the genomic and transcriptomic features of a rare breast malignancy, MPT. The molecular features of MPT presented here provide novel insights into the pathogenesis of this biphasic neoplasm showing both mesenchymal and epithelial components. Furthermore, by using a PDX model derived from the MPT, we demonstrated the possibility of driver and microenvironment roles for each component. Finally, we showed that targeting the PDGFR and PI3K/mTOR pathways can be an effective treatment option for patients with MPT.

Previous studies of gene expression profiles of phyllodes tumours have mostly focused on the molecular characterisation of MPT within a wide range of fibroepithelial neoplasm [6–8]. For example, Vidal et al. [66] profiled gene expression data of 75 fibroepithelial tumours, including 11 MPT, and demonstrated that the dysregulation of epithelial- or luminal-related genes such as *CLDN3*, *CLDN7*, or apoptosis- and angiogenesis-related genes such as *VIM* or *PIK3CA* can be key molecular characteristics of MPT. In contrast, the present study included only pathologically proven MPT to identify the molecular features that can classify malignant tumours with different clinical characteristics. Our data suggested that the expression patterns of genes involved in ECM interactions, EMT, and cell adhesion could classify MPT into two subtypes. The fact that the two subtypes of MPT are characterised by genes related to epithelial and mesenchymal differentiation indicated that the biphasic pathological features of fibroepithelial neoplasms are also reflected at the molecular level. Moreover, we showed that the molecular subtype of MPT is significantly associated with the degree of epithelial cell proportion, tumour size, and stromal overgrowth. We further inferred that the presence of mutations in *MED12* may be related to the molecular subtype of MPT. Notably, the *MED12* mutation, found in the stroma [10, 67], appears frequently in the group in which the epithelial component is relatively high. As this study had a small number of samples, further studies using sufficient MPT samples to prove this will need to be carried out in the future.

Sawhney et al. previously suggested that stromal growth in fibroepithelial tumours may be affected by the epithelium, showing that stromal mitosis is more prevalent in stroma close to the epithelium [68]. Tse et al. suggested the stromal-epithelial interaction and the crucial role of the epithelium in tumour progression of phyllodes tumours, showing that epithelial ER expression was inversely related to stromal mitotic count [69]. Since then, few studies have been conducted on the relationship between the epithelial and stromal components in fibroepithelial neoplasms. In most previous studies, each component was separated and analysed using methods such as laser capture microdissection to unveil the molecular characteristics of the epithelial and stromal components [10, 67]. In the present study, we showed the molecular characteristics and roles of each of the epithelial and stromal components through bioinformatic deconvolution using bulk RNA-seq data for the MPT PDX model, suggesting that this method can enhance the understanding of the pathogenesis of fibroepithelial neoplasms consisting of two components.

The genomic and transcriptomic findings in our study identified two druggable pathways in MPT: the PDGFR and PI3K/mTOR pathways. PDGFRα and PDGFRβ proteins are upregulated in stromal cells of human cancers [70] and are expressed in 10–70% of borderline or MPTs [71]. The stromal activity of PDGFR is associated with histologic grades and treatment outcomes of phyllodes tumours [52]. Moreover, the PI3K/mTOR pathway has been identified as a critical regulatory pathway in MPT [72, 73]. We showed that pharmacological inhibition of the PDGFR and PI3K/mTOR pathways with imatinib mesylate and PKI-587 effectively suppressed tumour growth in vivo. Our study shows targeted approaches that are viable therapeutic tools for the treatment of MPT. The proposed therapeutic approach should be tested in future clinical trials.

## Supplementary information

Supplementary figures and tables

Supplementary Data 1

Supplementary Data 2

## Data Availability

The data sets used and/or analysed during the current study are available from the corresponding author on reasonable request.
