# Supplementary material for: An integrative approach for exploring the nature of fibroepithelial neoplasms
Source: Br J Cancer. 2022 Dec 16;128(4):626–37. doi: 10.1038/s41416-022-02064-2 (PMC9938154; doi:10.1038/s41416-022-02064-2)
Supplement: Supplementary file 1 — Supplementary figures and tables [file 41416_2022_2064_MOESM1_ESM.pdf]

Supplementary figures and tables

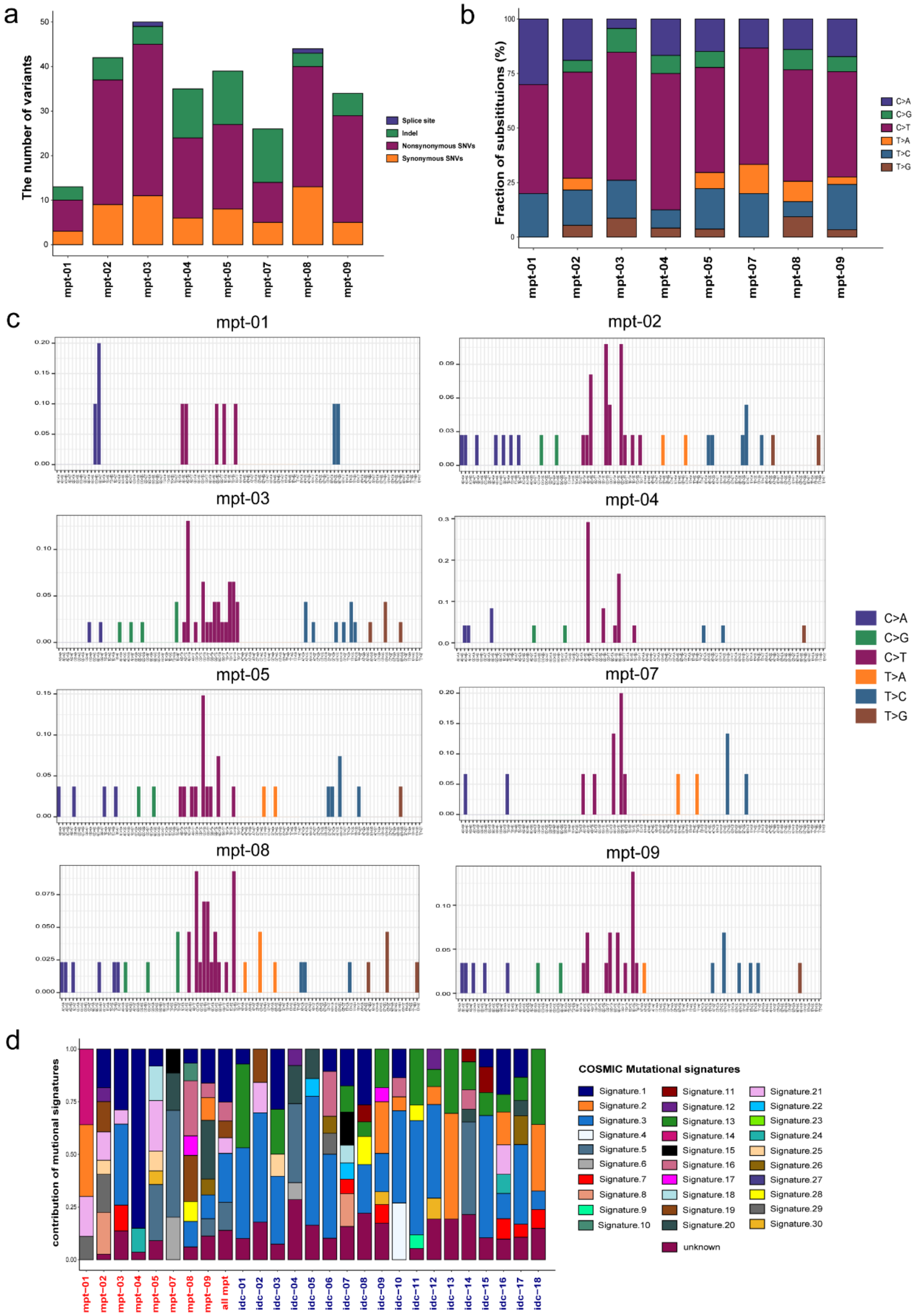

Supplementary Fig. S1: Mutational landscape of malignant phyllodes tumors (MPTs).

a Bar plot showing the burden of each type of somatic mutation.

b–d Mutational signatures in the MPT. Fraction of each nucleotide substitution **(b)** and fraction of variation in each trinucleotide context **(c)** in each sample of the MPT, and contribution of each mutational signature in MPT and invasive ductal carcinoma (IDC) **(d)** identified by R package 'deconstructSigs'. MPT samples are marked in red, and IDC samples are marked in navy in **(d)**. The "all mpt" in **(d)** shows the contribution of mutational signatures for whole substitutions in eight MPT samples.

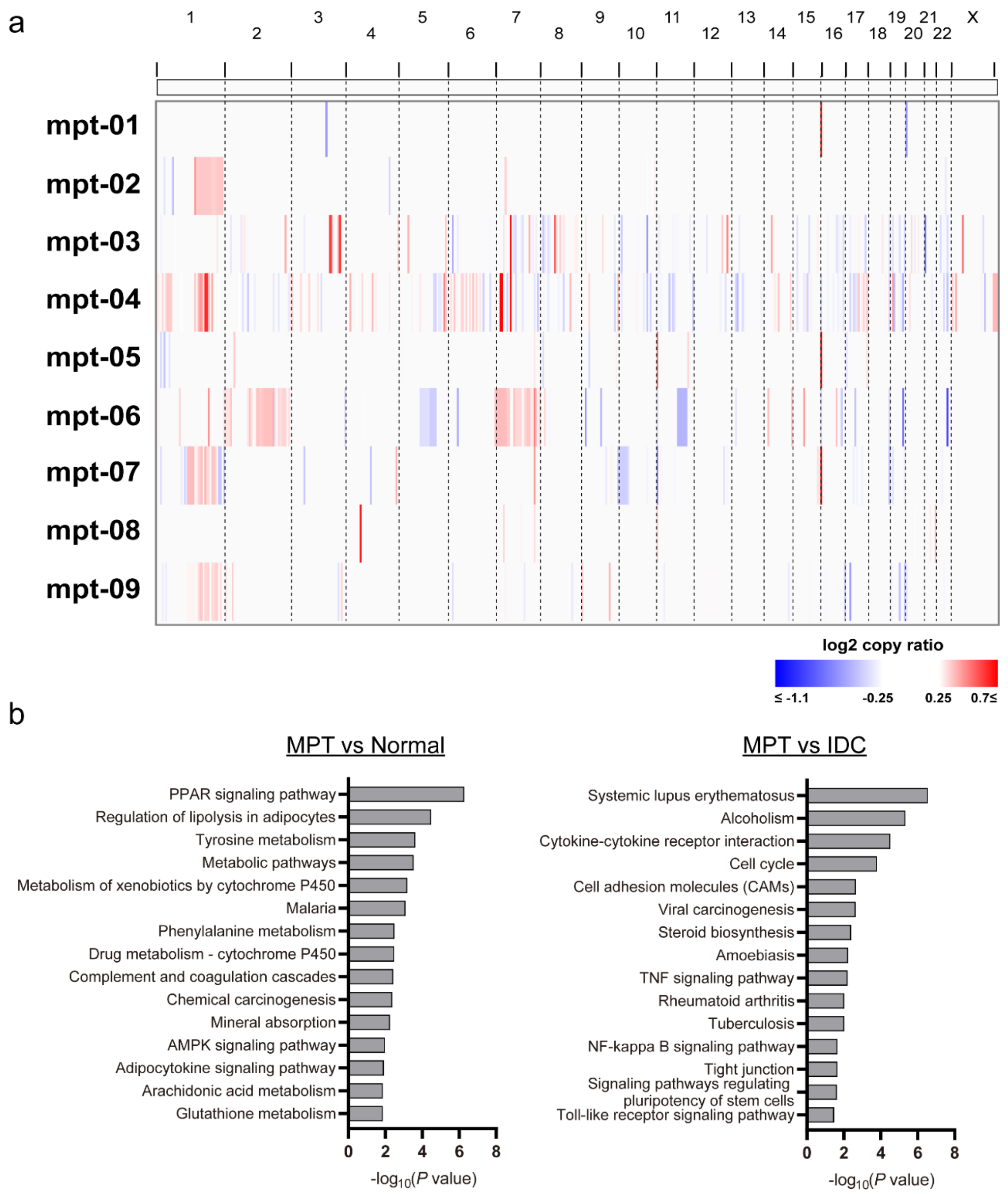

Supplementary Fig. S2: Genomic alterations and dysregulated KEGG pathways in nine malignant phyllodes tumors (MPTs).

a Genome-wide copy number changes in the MPT. Integrative Genomics Viewer (IGV) visualization of copy number variations identified using the R package ‘DNAcopy’.

b KEGG pathways enriched with downregulated genes in MPT compared to normal breast tissues (left) or invasive ductal carcinoma (IDC) tissues (right).

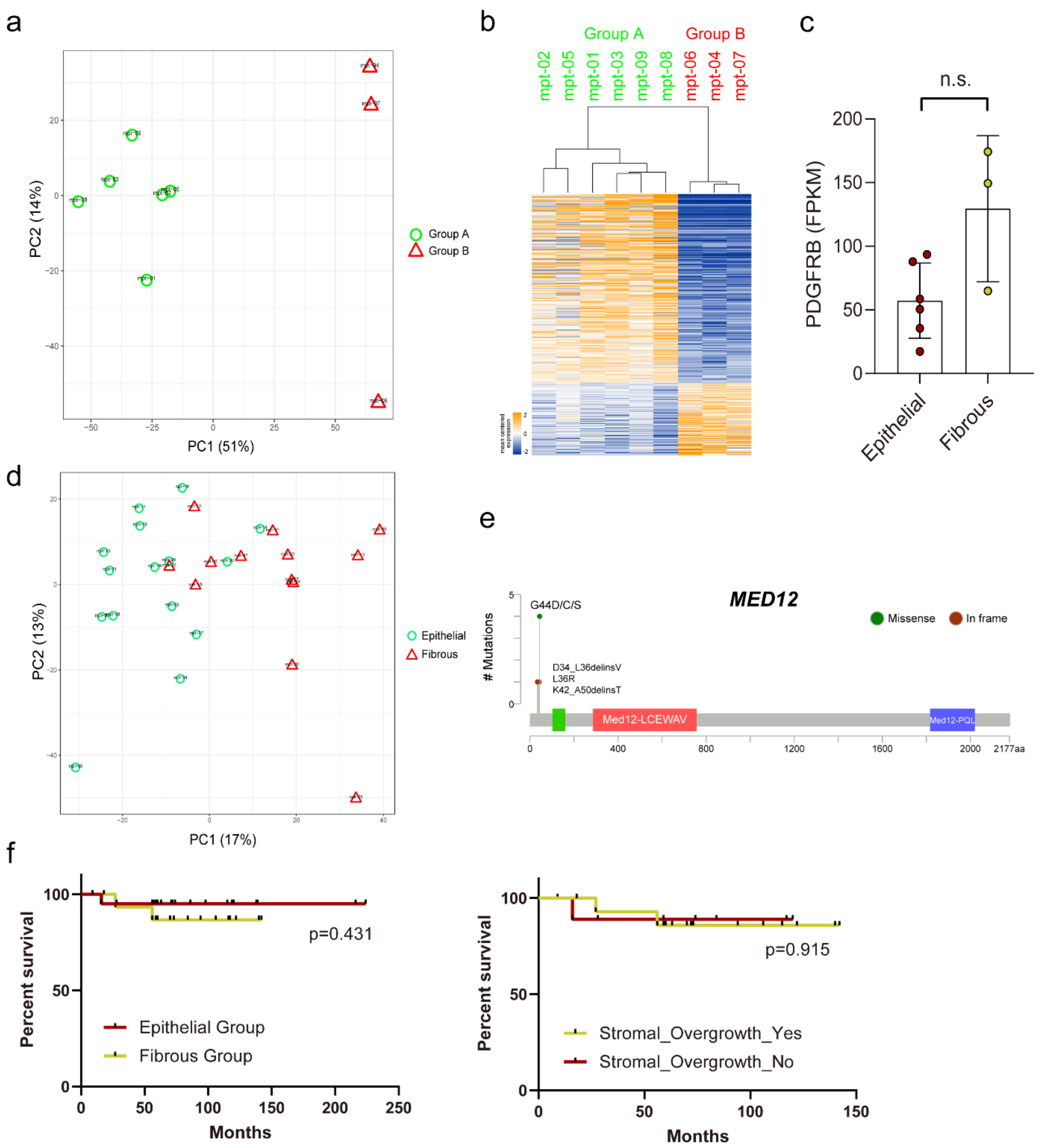

Supplementary Fig. S3: Two molecular subtypes of malignant phyllodes tumors (MPTs).

a Plot showing the first two PCs from principal component analysis (PCA) using most variable genes (n = 1000) of nine MPT fresh frozen (FF) samples.

b Heatmap showing the expression levels of differentially expressed genes (DEGs) between “Group A” and “Group B” of nine MPT.

c Expression levels of *PDGFRB* according to the molecular subtype in nine MPT (Mann-Whitney test). n.s.: not significant.

d Plot showing the first two PCs from principal component analysis (PCA) using most variable genes (n = 1000) of 28 MPT formalin-fixed, paraffin-embedded (FFPE) samples.

e Mutations in the *MED12* gene identified among the nine FF and 28 FFPE MPT are depicted using 'MutationMapper' provided by cBioPortal (http://www.cbioportal.org/mutation_mapper.jsp).

f Kaplan-Meier survival curves according to molecular subtypes of MPT (left) and histological stromal overgrowth (right) (log rank test).

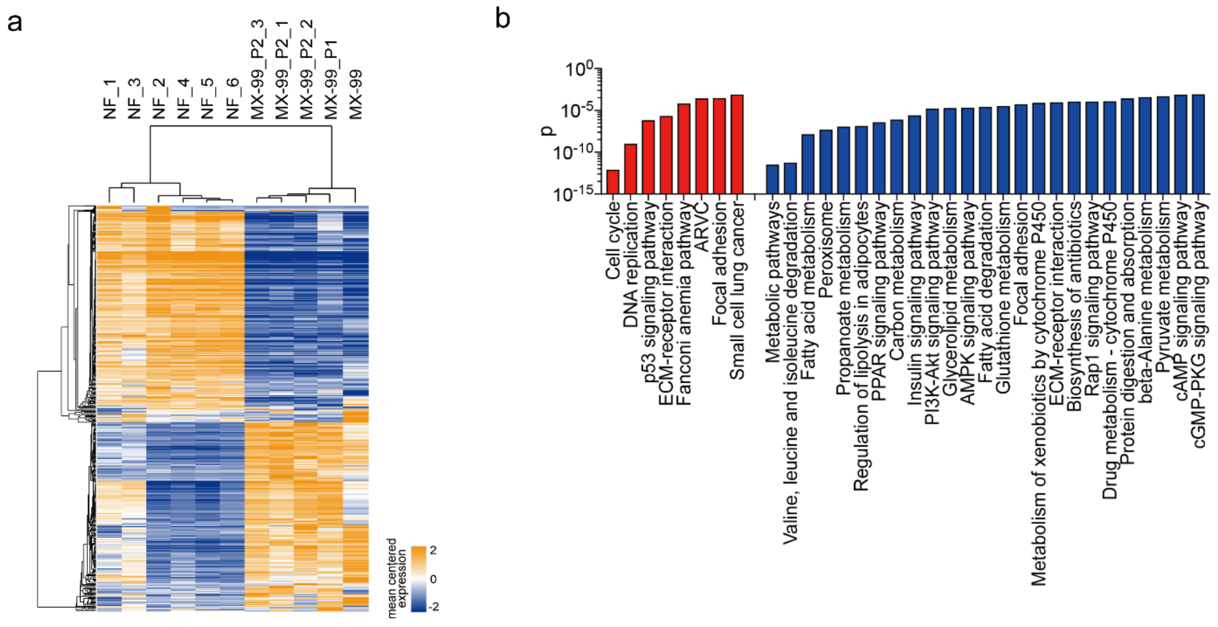

Supplementary Fig. S4: Changes of expression pattern in the microenvironment of malignant phyllodes tumors (MPTs).

a Unsupervised clustering of MPT and normal mouse fat pads using the expression levels of the top 2000 variable genes, except for genes with low expression values. (NF: normal mouse fat pad). P1 and P2 in the sample names indicate the PDX mouse passage number.

b Pathways enriched with upregulated genes (red) and downregulated genes (blue) in the murine microenvironment of MPT patient-derived xenografts (PDX) when compared to the mouse gene expression profile of normal mouse fat pads.

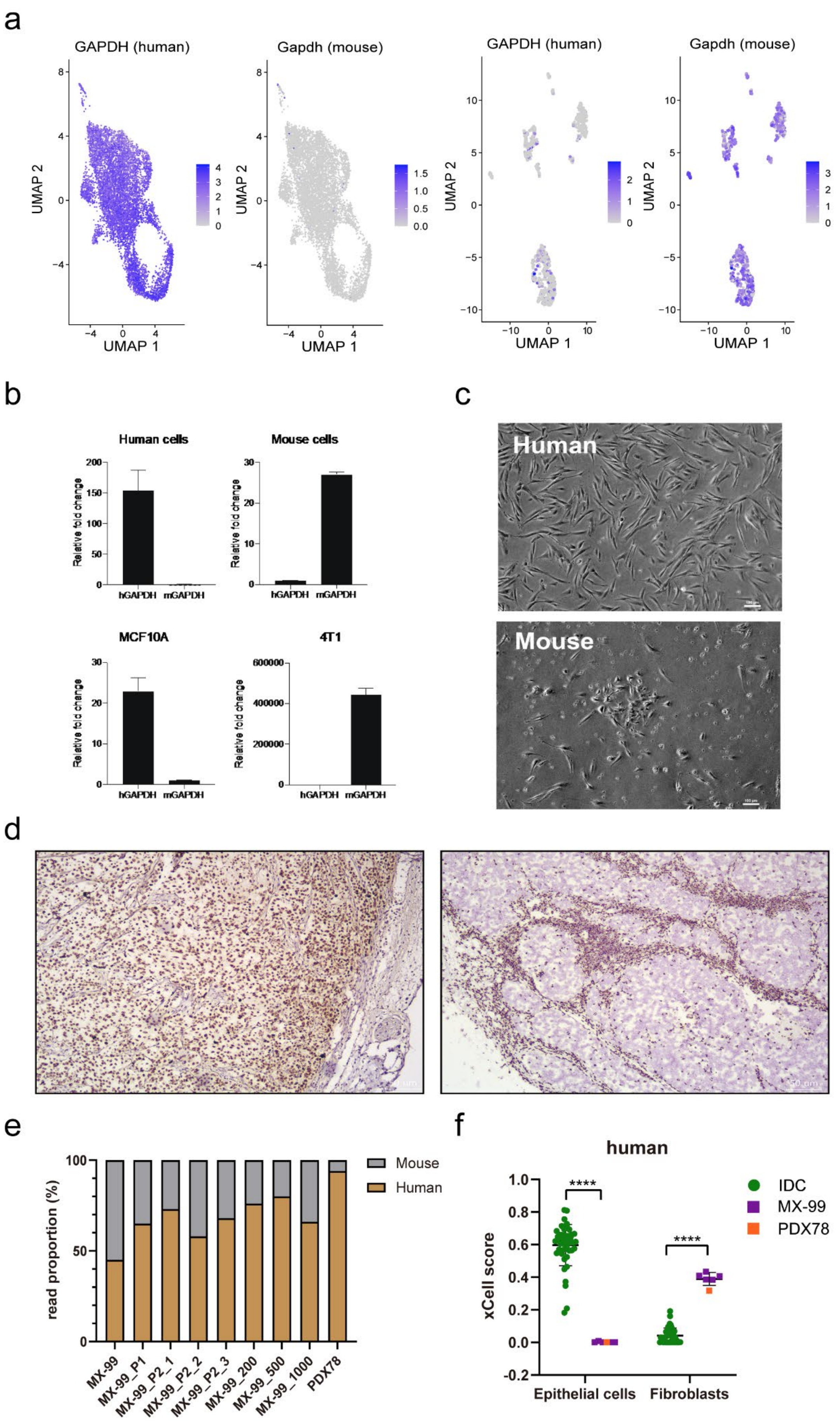
a
GAPDH (human)
Gapdh (mouse)
GAPDH (human)
Gapdh (mouse)
UMAP 1
UMAP 2
b
Human cells
Mouse cells
MCF10A
4T1
Relative fold change
hGAPDH
mGAPDH
c
Human
Mouse
d
e
Mouse
Human
read proportion (%)
MX-99
MX-99_P1
MX-99_P2_1
MX-99_P2_2
MX-99_P2_3
MX-99_200
MX-99_500
MX-99_1000
PDX78
f
human
xCell score
****
IDC
MX-99
PDX78
Epithelial cells
Fibroblasts

Supplementary Fig. S5: Tumor and microenvironment of malignant phyllodes tumor (MPT) characterization using the patient-derived xenograft (PDX) model.

a Cross-validation of the identity of grafted human cells (n = 8105) and mouse microenvironment (n = 858) using the housekeeping gene (GAPDH).

b Identification of human and mouse cell ratios by qRT-PCR using human-specific GAPDH and mouse-specific GAPDH primers in dissociated cells of MPT PDX tumors separated by MACS mouse depletion kit. Isolated human cells in MPT PDX tumor (top left), isolated mouse cells in MPT PDX tumor (top right), human mammary epithelial cell line MCF10A; positive control of human cells (bottom left), and mouse breast cancer cell line 4T1; positive control of mouse cells (bottom right).

c Cell morphology of dissociated MPT PDX tumor cells separated using the MACS mouse depletion kit. Isolated human (top) and mouse cells (bottom).

d Identification of human and mouse pan-centromeric probe FISH followed by anti-FITC immunostaining of the MDA-MB-231 xenograft tumor (left: human pan-centromeric probe, right: mouse pan-centromeric probe).

e Proportion of human and mouse RNA-seq reads in MPT PDX.

f Comparison of xCell scores of epithelial cells and fibroblasts using the human transcriptome data of invasive ductal carcinoma (IDC) PDX and MPT PDX (“MX-99” and “PDX78”) (Mann-Whitney test). ****: $p < 0.0001$

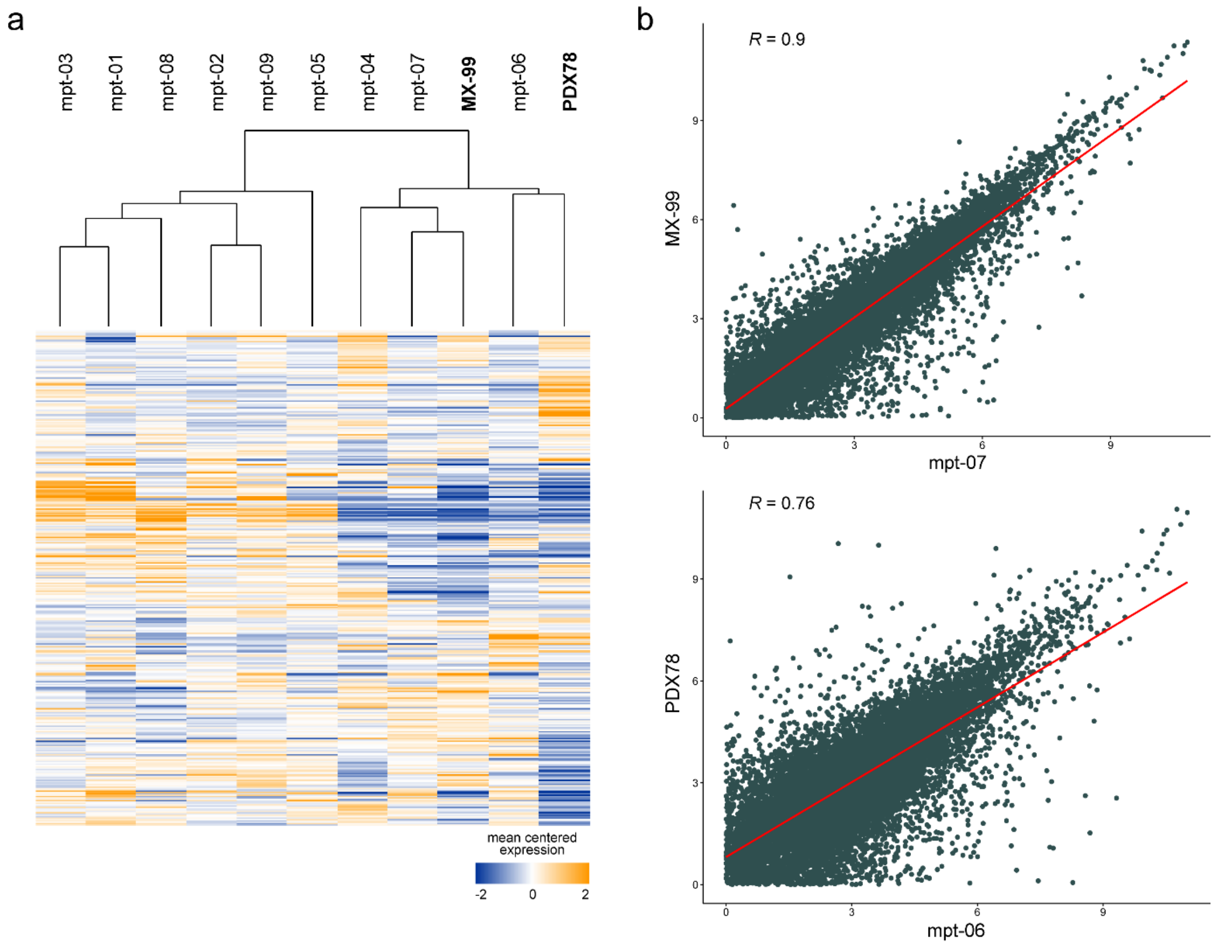

Supplementary Fig. S6: Transcriptomic similarity between the primary tumor of the patient and the PDX model.

a Hierarchical clustering of nine MPT patient tumors and two MPT PDX tumors using the expression levels of genes with an average FPKM value across all samples ≥1. MPT PDX tumor samples are indicated in bold.

b Comparison of the correlation between mRNA expression in the matched pair of patient tumor and PDX model (top) and the correlation between mRNA expression in the unmatched pair of patient tumor and PDX model (bottom).

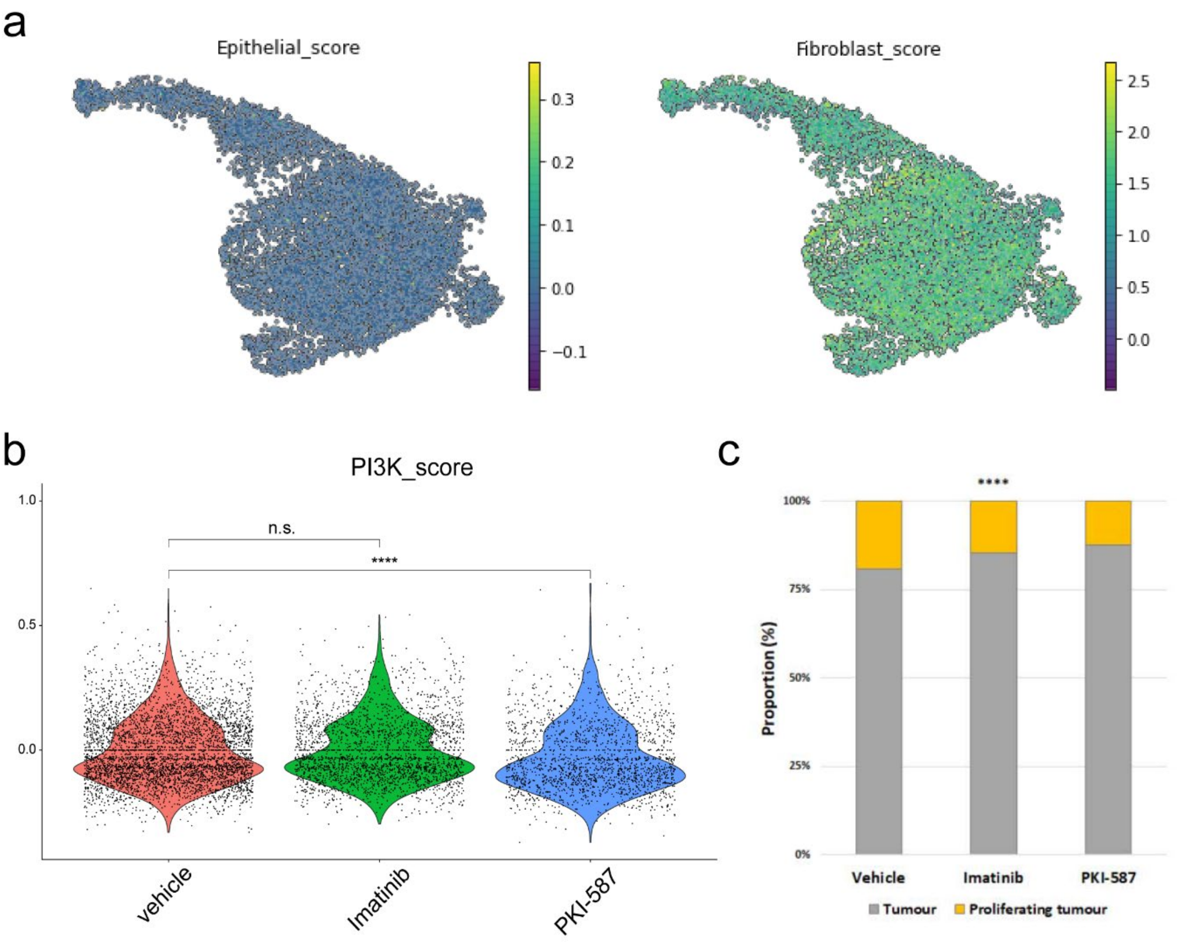

Supplementary Fig. S7: MPT PDX tumor cells treated with vehicle, imatinib, and PKI-587.

a UMAP plots of 8631 cells in the tumor population colored by epithelial and fibroblast scores. Epithelial and fibroblast cells were identified by the expression of marker genes. (Epithelial: *EPCAM, KRT14, MUC1, TP63*; Fibroblast: *FN1, VIM, COL1A1, COL6A2*)

b Comparison of PI3K pathway activity in tumor cells treated with vehicle, imatinib, or PKI-587 (Student's t-test). ****: p <0.0001.

c Relative cellular composition of tumor cells treated with vehicle, imatinib, and PKI-587 (chi-square test). ****: p <0.0001.

Sample ID	Depth of Coverage		Variants	
	Normal	Tumor	Total	Noteworthy
mpt-01	93.58	89.92	13	8
mpt-02	112.73	95.39	42	31
mpt-03	49.36	92.28	50	33
mpt-04	96.63	82.46	35	22
mpt-05	90.96	90.59	39	23
mpt-06	n/a	91.35	n/a	n/a
mpt-07	89.05	70.14	27	18
mpt-08	104.47	188.67	46	28
mpt-09	101.09	101.3	34	23

Supplementary Table S1: Information on the exome sequencing data.

The 'Total' indicates the number of variants that passed specific criteria among those called by MuTect and IndelGenotyper V2. The 'Noteworthy' indicates the number of variants that passed additional filters among 'Total' variants. Matched normal blood sample was not available for the sample "mpt-06," and therefore, the number of somatic mutations was not calculated.

Characteristics	Total (n=37)	Epithelial group (n=21)	Fibrous group (n=16)	P-value
Age	41.54±11.68	40.52±12.33	42.86±11.03	0.552
Stromal cellularity				0.389
High	20 (54.1%)	10 (47.6%)	10 (62.5%)	
Moderate	12 (32.4%)	7 (33.3%)	5 (31.3%)	
Low or none	2 (5.4%)	2 (9.5%)	0	
Unknown	3 (8.1%)	2 (9.5%)	1 (6.3%)	
Nuclear pleomorphism				0.384
High	13 (35.1%)	6 (28.6%)	7 (43.8%)	
Moderate	15 (40.5%)	7 (33.3%)	8 (50.0%)	
Low or none	5 (13.5%)	4 (19.0%)	1 (6.3%)	
Unknown	4 (10.8%)	4 (19.0%)	0	
Mitosis				0.623
>20	7 (18.9%)	3 (14.3%)	4 (25.0%)	
10~20	19 (51.4%)	10 (47.6%)	9 (56.3%)	
<10	9 (24.3%)	6 (28.6%)	3 (18.8%)	
Unknown	2 (5.4%)	2 (9.5%)	0	
Necrosis				0.08
Present	11 (29.7%)	2 (9.5%)	9 (56.3%)	
Absent	10 (27.0%)	6 (28.6%)	4 (25.0%)	
Local recurrence	9 (24.3%)	5 (23.8%)	4 (25.0%)	0.615
Distant metastasis	3 (8.1%)	1 (4.8%)	2 (12.5%)	0.396

Data are presented as number of patients (%) or mean ± standard deviation (SD).

Supplementary Table S2: Clinical information of the patients.
